# Inflammation and Epstein–Barr Virus at the Crossroads of Multiple Sclerosis and Post-Acute Sequelae of COVID-19 Infection

**DOI:** 10.3390/v15040949

**Published:** 2023-04-12

**Authors:** Beth A. Rousseau, Sumita Bhaduri-McIntosh

**Affiliations:** 1 Division of Infectious Diseases, Department of Pediatrics, University of Florida, Gainesville, FL 32610, USA; 2 Department of Molecular Genetics and Microbiology, University of Florida, Gainesville, FL 32610, USA

**Keywords:** Epstein–Barr virus, multiple sclerosis, COVID-19, post-acute sequelae of COVID-19 infection, PASC, inflammation

## Abstract

Recent studies have strengthened the evidence for Epstein–Barr Virus (EBV) as an important contributing factor in the development of multiple sclerosis (MS). Chronic inflammation is a key feature of MS. EBV^+^ B cells can express cytokines and exosomes that promote inflammation, and EBV is known to be reactivated through the upregulation of cellular inflammasomes. Inflammation is a possible cause of the breakdown of the blood–brain barrier (BBB), which allows the infiltration of lymphocytes into the central nervous system. Once resident, EBV^+^ or EBV-specific B cells could both plausibly exacerbate MS plaques through continued inflammatory processes, EBV reactivation, T cell exhaustion, and/or molecular mimicry. Another virus, SARS-CoV-2, the cause of COVID-19, is known to elicit a strong inflammatory response in infected and immune cells. COVID-19 is also associated with EBV reactivation, particularly in severely ill patients. Following viral clearance, continued inflammation may be a contributor to post-acute sequelae of COVID-19 infection (PASC). Evidence of aberrant cytokine activation in patients with PASC supports this hypothesis. If unaddressed, long-term inflammation could put patients at risk for reactivation of EBV. Determining mechanisms by which viruses can cause inflammation and finding treatments for reducing that inflammation may help reduce the disease burden for patients suffering from PASC, MS, and EBV diseases.

## 1. The Epstein–Barr Virus and Multiple Sclerosis: Connected, but How?

The Epstein–Barr virus (EBV) is a double-stranded DNA virus that is best known for causing infectious mononucleosis (IM). Less well-known, but arguably more important, EBV can lead to several cancers, including immunosuppression-associated post-transplant lymphoproliferative diseases/lymphomas, nasopharyngeal cell carcinoma, and Burkitt lymphoma [1,2]. EBV has also been linked to multiple sclerosis (MS), though causality has been difficult to demonstrate [3]. The difficulty in linking EBV and MS has been two-fold. First, EBV is present in upwards of 95% of the adult population. A gammaherpesvirus, EBV infects B and epithelial cells, but in B cells becomes permanent as the virus shifts into a latent state after acute infection [2,3]. Although EBV can reactivate into a lytic/productive phase, latency helps the virus evade host immune responses, allowing it to persist for the life of the host. With EBV nearly ubiquitous, evidence for a causal relationship to MS, a disease that is rare, has not been forthcoming. That said, dozens of studies point towards a possible contributory role for EBV [4,5,6,7]. The second challenge in connecting EBV to MS is temporal. MS is almost always an adult-onset neurodegenerative disease diagnosed between the ages of 20 and 40 [8]. With most individuals infected before 20 years of age, this leads to a central, unresolved question: how does EBV trigger MS after years or even decades of quiet latency?

MS is identified by demyelinating lesions in the brain detected through magnetic resonance imaging [9]. These lesions result in progressive loss of motor control, numbness or weakness of the limbs, visual impairment, and other symptoms that severely impact the quality of life. There are over a dozen FDA-approved disease-modifying therapies for MS, but no cure. Although a single disease, MS is heterogeneous in the age of clinical presentation, rate of disease progression, and suspected etiology. Indeed, genetics and environmental factors can both play a role. An environmental factor, EBV was recently shown to be linked to the development of MS through a study of over 10 million young active-duty US military personnel [10]. The risk of MS increased 32-fold after infection with EBV, but infection with other viruses did not show the same increased risk. Notably, serum levels of the neurofilament light chain, a biomarker of neuroaxonal degeneration, rose only after infection with EBV. Genetic susceptibility and the presence of a herpesvirus may be the driving force behind the development of MS for many patients. However, much of the molecular mechanisms underlying MS and drivers of pathogenesis remain to be elucidated. A number of mechanisms, including inflammation, molecular mimicry, dysregulation of B/T cell interactions, and activation of endogenous retroviruses, have been implicated [11]. These mechanisms, while not necessarily mutually exclusive, may singly or in combination explain the heterogeneity inherent to MS.

Inflammation is typically thought of as an acute process where the release of cytokines and chemokines leads to capillary dilatation, leukocytic infiltration, redness, heat, and pain. However, inflammation can also be chronic such as in MS. Inflammation in MS is characterized by infiltration of peripheral immune cells into the central nervous system (CNS), though whether this happens at the outset of the disease or secondary to a primary irritant is unknown. The discovery of EBV^+^ B cells in MS lesions suggests that they infiltrate the brain through a damaged blood–brain barrier (BBB) [12]. EBV-reactivated B cells could provide a mechanism for compromising the BBB through their activation of T cells, the release of exosomes harboring inflammatory viral RNAs and viral proteins, or through their own apoptosis, where they release viral products such as EBNA1, which further stimulate immune cells into action [11,13]. Alternatively, exosomes and cytokines that cross the intact BBB could stimulate astrocytes and microglia to cause inflammation which leads to porosity in the BBB and infiltration of EBV^+^ B cells or other immune cells that exacerbate the lesion [14,15]. Latent EBV^+^ B cells may also contribute to inflammation via cytokines such as TNF-α, TNF-β, and G-CSF, which are known to be upregulated in B cells transformed by EBV [16]. Late or early BBB breakdown likely worsens MS, and there is plausible evidence for both [11,13,14,15].

Although B cells are initially infected by EBV in the tonsils, they travel to other anatomical sites. If, in the case of MS, EBV^+^ B cells find their way into the CNS, possibly due to a breakdown in the BBB due to concurrent inflammatory processes, they could potentially transition into the lytic state [12]. Indeed, activation of the inflammasome has been found to be a cellular switch upstream of the viral lytic switch [17,18]. There is evidence for lytic EBV^+^ B cells occupying the CNS of MS patients [5]. Reactivation of EBV in B cells would normally be silenced through the destruction of infected B cells by T cells. However, T cell exhaustion may keep them from killing B cells harboring reactivated EBV. T cell exhaustion is characterized by a progressive loss of function, with the exhausted cells initially halting production of IL-2, then TNF-α, and finally IFN-γ [19]. Evidence for the exhaustion of EBV-specific CD8^+^ T cells in MS comes from a study by Pender et al. [20], who found that MS patients, compared to healthy controls, have decreased CD8^+^ T cell response (indicated by the production of IFN-γ, TNF-α, and IL-2) to EBV lytic, but not CMV lytic, antigens. Further, while CD8^+^ T cells directed against EBV-latent antigens were increased in MS patients versus EBV-seropositive healthy controls, MS patient-derived T cells expressed fewer cytokines indicating latent antigen-directed T cell exhaustion. During MS attacks, the populations of EBV-specific CD4^+^ and CD8^+^ T cells expanded, with increased cytokine production by latent-EBV antigen-specific CD8^+^ T cells. Over time, EBV-specific CD4^+^ and CD8^+^ T cells progressively declined, another indicator of T cell exhaustion. These findings have led to a clinical trial (NCT02912897) where EBV-specific cytotoxic T lymphocytes are being used as a therapeutic in an attempt to control EBV^+^ B cells that may be causing or exacerbating MS attacks. Inflammation and T cell exhaustion are examples of processes that could be concurrent and exacerbate MS.

Molecular mimicry is considered a leading mechanism underlying autoimmunity [21,22,23]. Molecular mimicry occurs when a foreign peptide elicits autoreactive B and T cells due to its resemblance to a self-peptide. New evidence from Lanz et al. [24] shows molecular mimicry between the EBV transcription factor EBV nuclear antigen 1 (EBNA1) and the CNS protein glial cell adhesion molecule 1(GlialCAM1), known to be expressed in chronic-active MS plaques [25]. Lanz et al. found that eight out of nine MS patients had monoclonal antibodies capable of binding peptides from the latent EBV protein EBNA1, highlighting the immunogenicity of EBNA1. An EBNA1 antibody derived from patient cerebrospinal fluid (CSF) was shown to also bind GlialCAM1 more tightly upon phosphorylation of GlialCAM1. The selection of antibodies with increased binding to GlialCAM1 is suspected to be the result of immature B cells encoding a germline precursor of a particular EBNA1 antibody entering the CNS, encountering GlialCAM1, and then undergoing affinity maturation resulting in clones encoding high-affinity anti-GlialCAM1 antibodies. Somatic hypermutation after the B cells have entered the CNS would require helper T cells and follicular dendritic cells to encourage the evolution of an EBNA1 targeting antibody into an antibody that also strongly recognizes GlialCAM1 [24,26]. This evidence for molecular mimicry is compelling, but this mechanism would require immune cells to have already infiltrated into the CNS, a process that might be better explained by inflammation.

Epidemiologic and molecular evidence supports a connection between EBV and MS in many cases. That said, it is not clear whether EBV^+^ or EBV-specific B cells initiate MS, but in either case, B cell infiltration likely occurs through the inflammatory breakdown of the BBB. After infiltrating the CNS, EBV^+^ B cells could become lytic and further contribute to inflammation. Conversely, EBV^+^ B cells could remain latent but continue to express cytokines or other factors that increase inflammation. There is still much to learn about EBV and MS, and it remains possible that different molecular mechanisms drive disease progression in different patients. Inflammation via the expression of cytokines [16], exosomes [14], or lytic reactivation [13,20] is a common thread that is not mutually exclusive in relation to molecular mimicry or T cell exhaustion.

## 2. PASC

There have been many reports on PASC (also called long COVID), a syndrome that is characterized by recovery from acute COVID infection but with symptoms that persist for weeks or months [27]. One of the most common symptoms of PASC is lingering fatigue [28]. The symptoms of PASC are not unique but rather commonly reported after a severe viral infection, such as from influenza, RSV, or even EBV [29,30,31]. What may be more unique about PASC versus other post-viral syndromes is that there has been an unprecedented effort to isolate molecular markers that identify or predict PASC. A cohort study of PASC patients found that plasma levels of c-reactive protein, D-dimer, and biomarkers of cardiac injury or dysfunction (troponin I, pro-B-type natriuretic peptide) and brain injury (neurofilament light chain) were similar between individuals who had versus had not had COVID-19 [32]. There is ongoing recruitment for an observational study where patients who have recovered from COVID-19 will be followed for 5 years to determine if demographics, length of illness, level of medical care, therapies received, or other factors may have an effect on PASC diagnosis and duration (NCT04964115). Recent reports have indicated that chronic inflammation may underlie PASC. The triad of IL-1β, IL-6, and TNF cytokines has recently been identified as a potential biomarker of PASC [33,34,35]. These cytokines are already associated with diseases such as rheumatoid arthritis and psoriasis (TNF), Castleman disease and rheumatoid arthritis (IL-6) [36], and type 2 diabetes, smoldering myeloma, urate crystal arthritis, and osteoarthritis among others (IL-1β) [37]. It is indeed plausible that the dysregulation of these cytokines contributes to PASC, and if so, already approved monoclonal antibodies could potentially be used as therapeutics to treat PASC [36].

If elevated cytokine levels contribute to PASC, their short half-lives necessitate that they are produced frequently or continuously by immune cells in order to remain elevated [38,39]. Monocytes and macrophages produce cytokines and are critical components of the inflammation and immune response [40]. There are three broad subpopulations of monocytes, classical (CD14^+^CD16^−^), intermediate (CD14^+^CD16^+^), and non-classical (CD14dimCD16^+^). Classical monocytes secrete pro-inflammatory molecules such as IL-6, IL-8, CCL2, CCL3, and CCL5. They also can differentiate into macrophages and dendritic cells, which aids in conversion from the innate immune response to the adaptive immune response. Intermediate monocytes are geared toward antigen presentation and secrete TNF-α, IL-1β, IL-6, and CCL3 upon TLR stimulation. Non-classical monocytes secrete TNF-α and are known to have a strong anti-viral response. A recent study by Ruenjaiman et al. has found that in recovered COVID-19 patients, reports of PASC symptoms one month post-infection are correlated with greater numbers of classical monocytes and an increase in the percentage of monocytes that are TNFα^+^ and IL-6^+^ compared to healthy controls [35]. The cohort with the greatest number of reported PASC symptoms also had a significantly reduced number of natural killer cells compared to healthy controls. The cohort size was small, but their data would suggest that hyperactivation of innate immune cells and a reduction in natural killer cells and possibly regulatory T cells contribute to PASC. Patterson et al. showed that the spike (S1) protein of SARS-CoV-2 could be found in CD16^+^ monocytes over one year post-infection [41]. Truncated, scant viral genomes would suggest that these S1 proteins are not from persistent infection but rather undegraded proteins from the original infection. Persistent viral antigens may contribute to PASC [41,42], but even in PASC patients not harboring viral antigens, intermediate and non-classical monocytes were still found to be elevated. Macrophages and T cells also produce cytokines [43]. Taken together, the immune cells that produce cytokines and the cells that are impacted by cytokines could become locked in a positive feedback loop where the cells continue to produce cytokines independently of the original stimulus.

Apart from cytokines and inflammation, there are other non-exclusive hypotheses for the cause of PASC under investigation [44]. As mentioned above, persistent viral antigens could promote inflammation or perhaps alter cell signaling and immune responses, although evidence for this remains limited [41,45]. The reactivation of endogenous retroviruses or resident exogenous viruses could also underlie PASC. In saliva samples taken from 19 individuals before and after COVID-19, IgM against HERV-K, and IgG antibodies against EBV, CMV, and HSV-1 all appear elevated post-infection [46]. EBV reactivation following COVID-19 will be discussed in more detail below. Dysregulation of the gut microbiome has been observed in PASC patients [47]. Tissue damage during COVID-19 infection may contribute to PASC [48,49]. Although early indicators point to inflammation as a molecular basis for PASC, more research is needed to examine all possible causes of PASC.

## 3. COVID-19 and Inflammation

**Figure 1:**
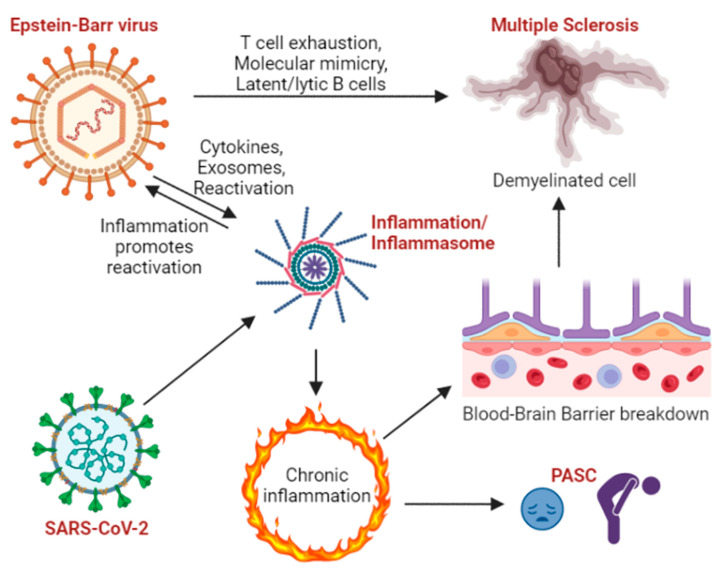
Inflammation at the intersection of EBV, MS, SARS-CoV-2, and PASC. EBV latent and lytic states can cause inflammation, and assembly/activation of the inflammasome can reactivate EBV. Neuronal demyelination contributes to MS, but EBV also contributes to MS through T cell exhaustion and molecular mimicry where latent or lytically infected B lymphocytes instigate immune dysregulation. SARS-CoV-2 activates the inflammasome and elicits inflammatory responses, which may trigger chronic inflammation, contributing to PASC. Chronic inflammation may also lead to BBB breakdown, which precipitates and/or exacerbates MS. Image created in BioRender.com accessed on 11 April 2023.

Inflammation is a key feature of COVID-19, with acute SARS-CoV-2 infection and exposure to viral components shown to strongly stimulate the NLRP3 inflammasome [50,51,52] (Figure 1). At the cellular level, activation of the NLRP3 inflammasome leads to the generation of IL-1β and sometimes pyroptosis [53,54]. The inflammasome is still a relatively underexplored pathway in the cell, and how inflammation is maintained over long periods of time has not been completely elucidated [55]. The inflammasome assembles when sensor proteins form a complex with ASC that activates caspase-1, which in turn activates IL1-β, IL-18, and gasdermin, ultimately mediating cell death through pyroptosis [55]. Sensors including NLRP3, AIM2, RIG-I, NLRP9b, IFI16, NLRP1, NLRC4, and NLRP12 can initiate this signaling cascade. Upon activation, the sensor binds to ASC, and the heterodimer oligomerizes into a large complex that then recruits and activates caspase-1.

It is possible that activation of the NLRP3 inflammasome by SARS-CoV-2 antigens may not lead to death in all cells but rather a persistent dysregulation of the NLRP3 inflammasome in a subset of cells that causes the symptoms associated with PASC. This is supported by the observation that some PASC patients exhibit elevated levels of IL-1β, which is the product of the active NLRP3 inflammasome [34]. Furthermore, there is some evidence that drugs that inhibit NLRP3 inflammasome activation improve outcomes in acute COVID-19 infections. Fluoxetine directly binds NLRP3 and blocks the formation of the NLRP3-Asc complex [56]. In a retrospective case-control study, fluoxetine use, in conjunction with other drugs, was associated with a 70% decrease in mortality among hospitalized COVID-19 patients [57]. Ibrutinib, used to treat chronic lymphocytic leukemia and other cancers, is an inhibitor of Bruton’s tyrosine kinase (BTK). Inhibition of BTK reduces the ability of NLRP3 to oligomerize and produce IL1-β [52,58]. If aberrant NLRP3 activation is a component of PASC, ibrutinib or a newer-generation BTK inhibitor may be able to reduce inflammation and alleviate symptoms. Metformin is another drug that has been demonstrated to inhibit NLRP3 inflammasome activation both in cell culture [52] and in an animal model [59]. Metformin is FDA-approved and prescribed as a treatment for type-2 diabetes. MCC950 is a specific inhibitor of NLRP3 which binds and prevents NLRP3 from hydrolyzing ATP and adopting or maintaining its active conformation [60]. In a mouse model of ulcerative colitis, MCC950 was shown to reduce inflammation, including IL1-β levels [61]. In human ACE2-transgenic mice infected with SARS-CoV-2, MCC950 was able to block NLRP3 activation and reduce the cytokine storm normally provoked by SARS-CoV-2 infection [62]. Given a possible role for inflammation in PASC, whether the therapeutic benefits of inflammasome-blocking agents also extend to PASC merits investigation

## 4. PASC and EBV Reactivation

A few small studies indicate that EBV reactivates in some individuals after SARS-CoV-2 infection [63,64,65,66]. Su et al. found that EBV reactivation in COVID-19 patients at the time of COVID-19 diagnosis was positively correlated with PASC symptoms of fatigue and sputum production 2 to 3 months after COVID-19 onset [66]. Chen et al. found that among a cohort of 67 SARS-CoV-2 infected patients, 37 were positive for IgM antibodies to EBV viral capsid antigen, indicative of either coinfection or EBV reactivation [63]. This 55.2% rate of EBV VCA IgM seropositivity in COVID-19 patients was higher than might be expected in the general population [67]. The patients with detectable anti-VCA IgM antibodies were three times more likely to manifest fever than patients without EBV reactivity, suggesting increased inflammation [63]. Gold et al. found that two-thirds of (20/30) PASC subjects demonstrated positive titers for IgG to EBV EA-D, suggestive of EBV reactivation in contrast to only 2 out of 20 control subjects with IgG antibodies to EA-D [64]. In a recent pre-print, Klein et al. detected increased antibodies against EBV small viral capsid antigen p23 and acute phase antigen EA-D in patients reporting PASC versus COVID-19 naïve controls and convalescent controls not reporting PASC [65]. As described above, SARS-CoV-2 is known to cause activation of the NLRP3 inflammasome [50,52], and EBV can be reactivated by activation of the NLRP3 inflammasome [18]. At face value, it seems plausible that SARS-CoV-2 infection could cause EBV reactivation in circumstances where EBV^+^ B cells are infected by SARS-CoV-2. However, the virus attaches to the ACE2 receptor, which is not expressed well in B cells [68]. Through trogocytosis, wherein cells can share their membranes and membrane-associated proteins with other cells, a B cell might be able to steal an ACE2 receptor and then become susceptible to SARS-CoV2 infection [69]. An alternate mechanism for the antigens of SARS-CoV-2 to enter B cells might be through exosomes secreted by cells that are infected by the virus [70]. It is further possible that reactivated EBV makes SARS-CoV-2 infections worse because it increases the expression of the ACE2 receptor [71]. Although inflammasome activation due to SARS-CoV-2 infection could lead to EBV reactivation, there could be other mechanisms by which EBV is reactivated, such as through the off-target effects of remdesivir [63], gut microbiota dysregulation leading to increased butyrate production [47], or XBP1 activation [72]. Certainly, more research is needed to better delineate the link between EBV reactivation and PASC.

## 5. Mechanisms for the Maintenance of Inflammation after EBV Reactivation, Acute COVID-19 Infection, or Multiple Sclerosis Onset

**Figure 2:**
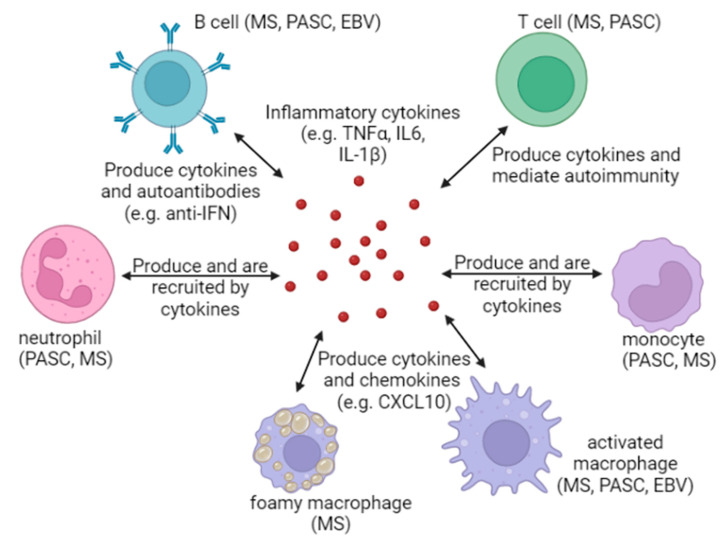
Innate and adaptive immune cells play a role in maintaining a state of inflammation frequently characterized by increased levels of cytokines. Some PASC patients have higher levels of neutrophils, which could promote an inflammatory state as they produce cytokines and are recruited by them. High levels of primed neutrophils have been observed in MS patients. Macrophages can have an anti-inflammatory effect early in MS, but as they become glutted with myelin, they can adopt a foamy, pro-inflammatory phenotype. Macrophages could also promote inflammation in MS via EBV exosomes. Macrophages may support inflammation in PASC through the production of cytokines and subsequent recruitment of more macrophages. Monocytes may have a pro-inflammatory effect in MS, especially by weakening the blood–brain barrier early in the disease. Monocytes in PASC patients may carry viral antigens long after the initial infection has resolved; higher numbers of pro-inflammatory classical monocytes were observed in some PASC patients early in recovery. T cells high in TNFα are carried by some PASC patients, which could provoke inflammation. In MS, T cells are known to recruit many other cell types into the brain and maintain a heightened state of inflammation that leads to plaque formation. B cells contribute to MS through molecular mimicry. Similarly, B cell production of autoantibodies to levels that are harmful could drive tissue damage and an increase in cytokines from damaged cells. B cells themselves are also known to produce cytokines. Image created in Biorender.com accessed on 11 April 2023.

As briefly touched upon earlier, immune cells are the mediators of inflammation, not simply the short-lived cytokines that they produce (Figure 2). Typical inflammatory cells include macrophages and neutrophils, which are the first line of defense against infections and produce the initial inflammatory response [73]. In tissues, neutrophils survive for only a few days, whereas macrophages can live for months. While neutrophils may not seem likely candidates for mediating a post-viral syndrome, follow-up of patients with severe COVID-19 disease has shown a neutrophil-associated immune signature [74]. An increase in the number of low-density neutrophils has also been found in PASC patients [75]. It is possible that neutrophils also play a role in MS, as neutrophils from MS patients have an increase in the surface receptors TLR2 and fMLPR compared to healthy controls [76,77]. Although the evidence is limited, neutrophils may contribute to a feedback loop that maintains heightened inflammation.

As discussed earlier, monocytes and macrophages may also contribute to continued inflammation in PASC. In MS, foamy macrophages can aid in the repair of demyelinated cells by removing damaged myelin which allows fresh myelin to be deposited on neurons [78]. However, prolonged consumption of myelin impairs the ability of foamy macrophages to conduct lipophagy, i.e., degrade lipids. This induces an inflammatory phenotype in macrophages, impairing the repair of MS lesions. Indeed, preserving the ability of macrophages to clear myelin and maintain an anti-inflammatory phenotype may help in repairing MS lesions.

With regard to EBV, exosomes containing EBV proteins, specifically nuclear antigen EBNA1 and latent membrane proteins LMP1 and 2A, are more abundant in patients with active RRMS (relapsing and remitting MS) compared to exosomes derived from healthy controls and non-RRMS patients [14]. Notably, monocyte-derived macrophages exposed to EBV antigen-laden exosomes express more CXCL10 than cells exposed to exosomes lacking EBV antigens. CXCL10 is a pro-inflammatory chemokine that attracts monocytes, eosinophils, T cells, and NK cells [79]. That said, it is not known whether EBV antigen-laden exosomes are a factor in inflaming macrophages early or late in MS, but either is plausible. Furthermore, dendritic cells may also contribute to inflammation in MS and PASC [38,80].

While monocytes, macrophages, and neutrophils are cells of the innate immune system and are believed to be key drivers of inflammation, adaptive immune cells such as T and B cells can also drive and react to inflammatory cytokines. Patients with respiratory PASC were shown to have up to 105-fold higher frequencies of IFN-γ- and TNF-α-producing SARS-CoV-2-specific CD4^+^ and CD8^+^ T cells in peripheral blood and elevated levels of plasma IL-6 [81]. Importantly, SARS-CoV-2 specific T cell frequencies correlated positively with decreased lung function as measured by pulmonary function tests. The half-life of SARS-CoV-2-specific T cells is thought to be between 3 to 5 months, but it is possible that this population could persist or be maintained longer due to a persistent viral antigen or due to reinfection with SARS-CoV-2 before the T cells have been retired. In MS, T cells are known to be the drivers of disease due to their attack on myelin and recruitment and activation of other immune cells such as B cells, macrophages, dendritic cells, and microglia [9]. B cells may also contribute to MS through molecular mimicry, as mentioned earlier. Interestingly, autoantibodies may also be a mechanism through which PASC could be driven by B cells. Using rapid extracellular antigen profiling (REAP), a group at Yale University detected enrichment of autoantibodies against IL-6 in patients with severe and moderate COVID-19 compared to people with asymptomatic infection or no infection [82]. Autoantibodies against cytokines are known to interfere with their function. A collaborative effort using a multi-omics longitudinal approach discovered that in 309 COVID-19 patients, there was a positive correlation between levels of autoantibodies and levels of certain cytokines. For example, autoantibodies against IFN-α2 predicted higher plasma levels of IL6, CXCL10, and IFN-γ [66]. Mechanistically, B cells producing antibodies against cytokines could be dysregulating IFN-dependent responses. [83]. Monocytes from MS patients undergoing IFN-β treatment make substantially less IL-1β than monocytes derived from healthy donors, which implies that anti-IFN autoantibodies may contribute to an increase in inflammatory cytokines produced by monocytes [84]. In a study of 987 patients hospitalized for severe COVID-19 pneumonia, 13.7% were found to have IgG autoantibodies against at least one type I interferon. Although patient samples were not screened for molecular markers of inflammation, it is plausible that a deficient interferon response, either due to genetic mutations or autoantibodies, could predict high levels of inflammation [85,86]. There is early evidence that both innate and adaptive immune cells could contribute to maintaining inflammation. However, more research is needed to understand if these mechanisms are common to all PASC patients or only a subset of PASC patients and whether different MS patients may have different sets of immune cells driving their disease progression.

## 6. Inflammation: A Common Thread That Binds EBV, MS, and PASC

While inflammation is a complex process that affects cells specific to particular diseases, it also influences the microenvironment. IL-1β, IL-6, and TNF are three cytokines implicated in PASC and other inflammatory diseases such as rheumatoid arthritis, with IL-1 also contributing to inflammation in MS [87]. Moreover, while future studies might reveal if PASC is a heterogeneous disorder, the same as MS, treatments for both diseases may hinge on lowering inflammation and possibly controlling and maintaining the quiescence of EBV. Future studies need to focus on identifying biomarkers for PASC, determining whether anti-inflammatory treatments are empirically effective in treating both MS and PASC and examining EBV loads and EBV antibody titers before and after PASC diagnosis. Given the involvement of the NLRP3 inflammasome in MS, COVID-19, and reactivation of EBV from latency, the inflammasome may emerge as a common therapeutic target. There are already known drugs that target the NLRP3 inflammasome and others that are under development [53,88,89]. Beyond PASC, addressing inflammation caused by EBV^+^ B cells alone may ameliorate the pathogenicity and manifestations of MS. In summary, the emergence of new diseases such as PASC, improved understanding of links between MS and EBV, and the recognition of inflammation at the intersection of PASC, MS, and EBV underscore the need for more research in these areas to meet the needs of patients with MS, PASC, and EBV diseases.
